# What Are the Experiences, Views and Perceptions of Patients, Carers and Clinicians of Glucagon‐like Peptide‐1 Receptor Agonists (GLP‐1 RAs)? A Scoping Review

**DOI:** 10.1111/hex.70251

**Published:** 2025-04-14

**Authors:** Sam Febrey, Michael Nunns, Jill Buckland, Rebecca Abbott, Alison Bethel, Rebecca Whear, Kate Boddy, G. J. Melendez‐Torres, Jo Thompson Coon, Liz Shaw

**Affiliations:** ^1^ Isca Evidence, University of Exeter Medical School, Faculty of Health & Life Sciences, University of Exeter St Lukes Campus, Exeter Devon UK; ^2^ NIHR Applied Research Collaboration South West Peninsula (PenARC), University of Exeter Medical School, University of Exeter, St Lukes Campus, Exeter Devon UK

**Keywords:** diabetes, glucagon‐Like peptide 1 receptor agonists (GLP‐1 RAs), obesity, qualitative, scoping review, weight loss

## Abstract

**Background:**

Glucagon‐like peptide‐1 receptor agonists (GLP‐1 RAs) are a pharmacological treatment option for both diabetes and weight loss. Qualitative evidence is vital in providing greater understanding of patients, practitioners and carers experience of taking or delivering GLP‐1 RAs. This evidence can inform the current or future configuration and delivery of services. We conducted a scoping review to better understand the quantity, nature and key characteristics of qualitative primary evidence which explores the experiences, views and perceptions of patients, carers and clinicians regarding the use of GLP‐1 RAs.

**Methods:**

Four bibliographic databases were searched on 10 July 2024: MEDLINE, APA PsycInfo via Ovid, CINAHL Ultimate via EBSCOhost, ProQuest Dissertations and Theses Global via ProQuest. We also searched Google Scholar, two clinical trials registries, the pre‐print server medRxiv and conducted citation searches. We sought qualitative research about the experiences of patients, carers and practitioners about any aspect of taking or prescribing GLP‐1RAs, for any indication. Study selection and data extraction were performed by two independent reviewers. The included studies were collated, and their characteristics were described.

**Results:**

After de‐duplication 1545 titles and abstracts were screened for relevance, with 77 full‐text articles assessed for eligibility, resulting in 25 included studies. More studies were focused on type 2 diabetes (*n* = 12) than weight loss (*n* = 9) or any indication (*n* = 4). The experiences of carers were not represented. No one area of experience (e.g. different indications or viewpoints) was well represented, either due to the absence or narrow focus of studies or lack of an in‐depth analytical approach.

**Conclusion:**

Whilst primary qualitative evidence exploring patient and clinician experience of GLP‐1 RAs was identified in this scoping review, the findings highlight a need for more robust qualitative research to be conducted across all user groups, in particular involving carers, and especially for the indication of weight loss within service settings. This evidence gap needs to be urgently addressed to ensure GLP‐1 RAs are appropriately prescribed and patients and carers receive support from services suited to their needs.

**Patient or Public Contribution:**

Seventeen public collaborators contributed to the search by suggesting additional search terms, helping define the population for inclusion and contributing to protocol development. Their thoughts on the findings of the review helped form the basis for the discussion of this paper.

## 1 Introduction

Globally the number of overweight or obese people is increasing, with adult obesity doubling since 1990, leading to an estimated five million deaths from weight‐related health conditions such as cardiovascular diseases, diabetes and cancer [1], with growing concern regarding obesity‐related conditions in lower and middle‐income countries [2].

Glucagon‐like peptide‐1 receptor agonists (GLP‐1 RAs), such as semaglutide and liraglutide, and the dual gastric inhibitory polypeptide (GIP) and GLP‐1 RA, tirzepatide, are drugs authorised in the UK by NICE (National Institute for Health and Care Excellence) for the management of obesity and/or type 2 diabetes mellitus (T2DM) [3, 4, 5]. A recent systematic review which summarised and critically appraised evidence arising from existing network meta‐analyses (NMAs) evaluating the effectiveness of GLP‐1 RAs for weight loss in obese patients highlighted that of these three types of GLP‐1 RAs, subcutaneous semaglutide 2.4 mg and tirzepatide 15 mg were associated with the largest effects for weight loss at timepoints of six, 12 and 12+ months when compared to placebo [6]. Whilst tirzepatide and semaglutide were the most effective of these drugs for weight loss, they were also generally associated with increased risk of safety issues [6]. The review also identified the need for longer‐term trials to establish the efficacy and safety of GLP‐1 RAs when taken for longer than 72 weeks [6].

Qualitative research provides the opportunity to gain insight into perceptions of the long‐term efficacy, safety and acceptability of GLP‐1 RAs, as well as the benefits and challenges associated with introducing this type of intervention for patients and health services [7]. One systematic review explored patient and staff views of barriers and facilitators to initiating, and adhering to, injectable treatments for type 2 diabetes [8]. Only two of the 42 included studies included participants with experience of GLP‐1 RAs, but neither of these explored views of that specific treatment. Another systematic review searched for quantitative and qualitative evidence to explore ‘values, preferences and burden of treatment for the initiation of GLP‐1 receptor agonists and SGLT‐2 inhibitors in adults with Type 2 diabetes’ [9]. Whilst the results from this review were primarily based on GLP‐1 RAs, the findings were descriptive and do not provide an in‐depth understanding of the experiences of patients and/or staff of receiving or providing a prescription for GLP‐1 RAs. Scoping searches indicate the presence of a small body of primary research which focuses on the views and/or experiences of patients or clinicians on the use of GLP‐1 RAs to help control diabetes and/or promote weight loss. However, given the rapidly developing research landscape pertaining to this topic and changes in how these types of medications are prescribed and delivered, it is not certain the extent to which this existing evidence base is useful to inform the current or future configuration and delivery of services.

### 1.1 Aim

To conduct a scoping review to better understand the quantity, nature and key characteristics of primary evidence which explores the experiences, views and perceptions of patients, carers and clinicians regarding the use of GLP‐1RAs for any indication.

### 1.2 Research Question

What is the quantity, nature and key characteristics of primary evidence which explores the experiences, views and perceptions of patients, carers and clinicians regarding the use of GLP‐1 RAs for any indication?

## 2 Methods

Following identification of a research aim/question, there are five recognised stages to undertaking a scoping review: (i) identifying the relevant studies; (ii) study selection; (iii) charting of the data; (iv) collation, summarising and reporting the results and (v) consultation [10, 11]. We describe how our methods align to each of these stages below.

### 2.1 Search Methods

The search strategy was developed by an information specialist (J.B.), peer‐reviewed by a second information specialist (A.B.), in MEDLINE and translated to the other databases. The searches used a combination of relevant controlled vocabulary terms (e.g. Medical Subject Headings, MeSH) and free text terms. Validated search filters for qualitative studies were used for searching Medline [12], APA PsycInfo [13] and CINAHL Ultimate [14]. The search strategies are shown in Appendix A and the search summary table in Appendix B.

Four bibliographic databases were searched on 10 July 2024: MEDLINE (1946 – current), APA PsycInfo (1806 – current) via Ovid, CINAHL Ultimate via EBSCOhost, ProQuest Dissertations & Theses Global (1637 – current) via ProQuest. In addition, searches were conducted on Google Scholar (https://scholar.google.co.uk/) to identify unpublished reports. The databases were searched from inception to July 2024 with no date or language restrictions.

Searches for unpublished studies were carried out in two clinical trials registries and the pre‐print server, medRxiv (https://www.medrxiv.org/search). The clinical trials registries searched were International Clinical Trials Registry Platform (ICTRP) (https://www.who.int/clinical-trials-registry-platform) and clinicaltrials.gov. Results were downloaded into Endnote (version 20, Clarivate Analytics, Philadelphia, PA, USA) which was used for finding and removing duplicates. Forward and backward citation searching of included references was conducted in Scopus (1788 – present). Finally, potentially relevant systematic reviews that had been identified by searching were hand‐searched for includable studies. The citation searching results were downloaded into Endnote and de‐duplicated against the database search results.

#### 2.1.1 Inclusion Criteria

The following inclusion and exclusion criteria according to PICo (Population, phenomenon of Interest, Context) framework were applied. We sought the views of patients, carers or clinicians of any age, with experience of GLP‐1 RAs, whether this was being prescribed GLP‐1 RAs, supporting patients who have been prescribed them, prescribing them to patients, or supporting patient access to, or services delivering them. The drugs could be prescribed for any indication, in any healthcare or community setting. We excluded studies capturing the views of other treatments, where the experiences of GLP‐1 RAs could not be separated. Only qualitative or mixed‐methods primary studies were eligible. This included data captured with interviews and focus groups and analysed using qualitative methods (e.g. thematic analysis, framework analysis). We also included surveys or questionnaires using open questions or analyses of social media posts, so long as qualitative analysis techniques were used (e.g. content, thematic analysis). Trial registry items or protocols with clearly specified qualitative component were included. There were no restrictions on date or geographical area, but we only included English language studies, to avoid misinterpretation. Conference abstracts, systematic or narrative literature reviews and case studies were excluded.

#### 2.1.2 Study Selection

Two reviewers (S.F., M.N.) independently applied the inclusion and exclusion criteria to the titles and abstracts of records identified by the search. Disagreements were highlighted and consensus met by discussion. The full text of each paper was assessed in the same way. Endnote (version 20, Clarivate Analytics, Philadelphia, PA, USA) software was used to support study selection.

#### 2.1.3 Data Extraction

Descriptive data was extracted for each study by one reviewer and checked by a second (S.F., M.N., R.A.). This data included: first author, date of publication, title, focus/aim, country data collected within, conflict of interest, funding source, population characteristics (experience relating to GLP‐1 RAs, health conditions, age, gender, ethnicity, socioeconomic status, education, occupation, indication for GLP‐1 RAs), sample size, data collection setting, data collection technique (e.g. survey, interviews, focus group), type of analysis performed, themes or concepts presented relevant to the research question.

#### 2.1.4 Collation, Summarising and Reporting of Results

Key characteristics of all included studies were tabulated and described narratively. Studies conducted with similar population groups, examining similar phenomenon of interest were grouped together. The main findings are presented alongside a visual representation of the studies included in the review.

#### 2.1.5 Patient and Public Involvement and Engagement

This review benefited from collaboration with PERSPEX, a group of 17 public collaborators who bring their carer, patient, or public perspective to the work of Isca Evidence. PERSPEX members meet monthly online, and membership is culturally, geographically and demographically diverse (https://www.exeter.ac.uk/research/groups/medicine/esmi/workstreams/perspex/).

PERSPEX has been previously involved in two related reviews evaluating the effectiveness of GLP‐1 RAs for weight loss. Over the course of 12 months, PERSPEX members and researchers discussed GLP‐1 RAs at six online meetings and through various written and video material communications. These discussions highlighted safety as a key area of interest to patients and carers and contextualised the use of these drugs within a broader societal background, surfacing concerns about industry sponsorship. These discussions foregrounded patient, and carer matters for the review team and informed preliminary scoping work to define the research question for this review.

In this scoping review, PERSPEX checked the initial search strategy and made suggestions for additional search terms. A plain English version of the draft protocol was sent to members to read before the June 2024 online PERSPEX meeting. This document informed discussions about the review, particularly the included population, and whether this should include only those prescribed GLP‐1 RAs or those taking them through other means. This directly informed development of the review protocol. The preliminary findings of the review were discussed at the September 2024 online meeting.

## 3 Results

### 3.1 Study Selection

**Figure 1:**
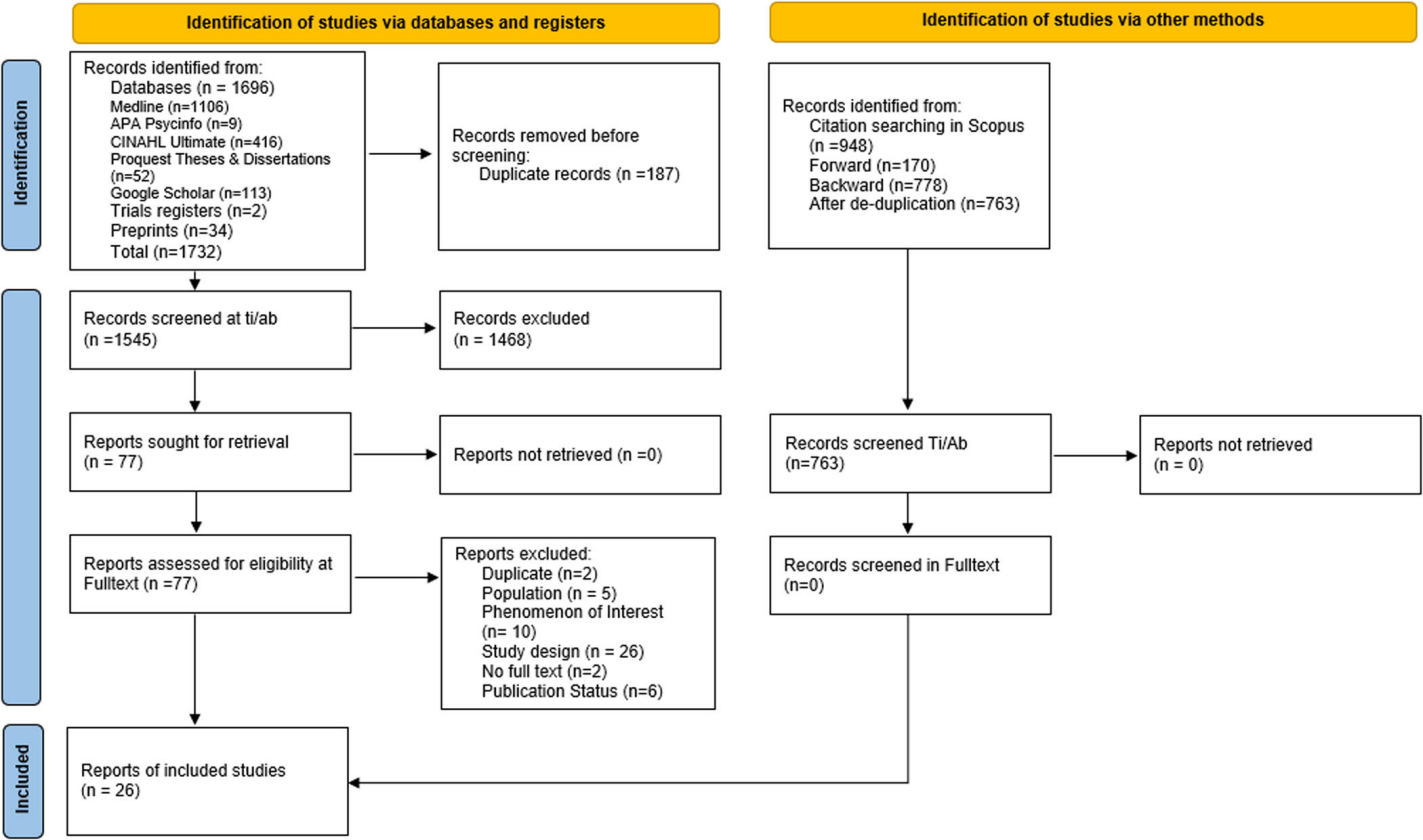
PRISMA flowchart summarising the results of the literature search and study selection.

The initial searches identified 1732 records. After de‐duplication 187 records were removed, and 1545 titles and abstracts were screened for relevance. After excluding 1468 records, a total of 77 full‐text articles were assessed for eligibility resulting in 25 studies reported in 26 reports. One study was reported in two papers [15, 16]. This is described in more detail in the study characteristics section below. The most common reasons for exclusion at the full‐text stage were study design (*n* = 26 reports) and phenomenon of interest (*n* = 10 reports). See Appendix B for the search summary table and Appendix C for a list of studies with reasons for exclusion at full text. The Preferred Reporting Items for Systematic reviews and Meta‐Analyses (PRISMA) flow diagram is shown in Figure 1.

### 3.2 Study Characteristics

Tables 1 and 2 display the study characteristics and data collection methods of the 25 included studies (from 26 reports) [16, 17, 18, 19, 20, 21, 22, 23, 24, 25, 26, 27, 28, 29, 30, 31, 32, 33, 34, 35, 36, 37, 38, 39, 40].

**Table 1:** Study characteristics of included studies.

Study ID	Country	Population/Data source	Experience relating to GLP‐1 RAs	Inclusion criteria	Sample size	Mean age, % female	Ethnicity and Race	Conflict of interest (Y/N/NR)	Funding
Alvarez‐Mon et al. [17]	Spain	Public tweets	Tweets related to GLP‐1 RAs of interest	Twitter: Hashtags on Xenical, orlistat, Alli, Belviq, lorcaserin, Qsymia, phentermine‐topiramate, Contrave, bupropion‐naltrexone, Saxenda, Victoza, liraglutide, Ozempic and semaglutide between September 20, 2019, and October 31, 2019; and posted in English language	Total tweets 780, 382 tweets linked to GLP‐1 RAs (Liraglutide and Semaglutide)	NR	NR	NR	Grants from the Fondo de Investigación de la Seguridad Social
Andreassen et al. [18]	Denmark	Clinicians	Prescribers and/or management of patients taking WeGovy (Semaglutide)	GPs in areas with a high prevalence of obesity, GPs recruited in terms of variation in location (town and country) and personnel (sex, age and seniority).	3 GP practices, 11 HCPs interviewed (6 GPs, 4 nurses, 1 junior doctor), 273 consultations observed (27 on WeGovy)	NR	NR	NR	Novo Nordisk Foundation
Arillotta et al. [19]	Italy	Public social media posts	Social media posts related to GLP 1RAs and mental health, and substance use/addiction	Reddit, Tik‐Tok and YouTube posts from December 2019 to June 2023 with the following keywords: Ozempic, semaglutide, tirzepatide, Mounjaro, Wegovy and Rybelsus	12,136 Reddit posts, 14,515 YouTube videos and 17,059 TikTok videos	NR	NR	Y	University of Hertfordshire
Arillotta et al. [20]	Italy	Public social media posts	Social media posts related to GLP 1RAs and substance use/addiction	Reddit, Tik‐Tok and YouTube posts from December 2019 to June 2023 with the following keywords: Ozempic, semaglutide, tirzepatide, Mounjaro, Wegovy and Rybelsus	5859 threads and 12,136 related comments were extracted from six subreddits.	NR	NR	Y	University of Hertfordshire
Barnard‐Kelly et al. [21]	UK	Patients and HCPs	Intervention arm of trial taking 3 mg injections of Liraglutide (trial)	Patients: (aged 21–64 years) overweight/obese with a clinical diagnosis of schizophrenia, schizoaffective disorder or first‐episode psychosis and treated with antipsychotic medication. HCPs: involved in the delivery of the intervention within the study	17 patients and 2 HCPs	Patients: 42.6 years, 38% HCPs: NR	Patients: white British and Irish 88%, other ethnicity 13% HCPs: NR	NR	Novo Nordisk Ltd and NIHR
Bradley et al. [22]	USA	HCPs	GLP1‐1RAs as one of the medications to consider in deprescribing/managing T2DM	Lifestyle medicine providers with experience treating T2DM with a goal of remission and supervising medication deprescribing	9	NR	NR	N	Ardmore Institute of Health
Bremmer et al. [23]	USA	Public social media posts	Public forums specific to use of alcohol and GLP‐1 RAs	Posts from three popular Reddit forums on Semaglutide, Mounjaro and Ozempic (each of which had more than 20,000 users) referring to alcohol use whilst using one of these medications	1557 posts	NR	NR	NR	NR
Ciemins et al. [24]	USA	HCPs	Prescriber for, or management of, patients with T2DM with/or at risk of CVD, eligible for GLP‐1 RAs	HCPs from 4 Health Care Organisations were selected on the basis of interest and willingness to participate	20	NR	NR	Y	Novo Nordisk
Donnan et al. [25]	Canada	Patients	Experience of taking GLP‐1 RAs in relation to weight management	English‐speaking Canadian residents ≥ 18 years, who had self‐reported obesity, with adiposity‐related complications	21	76.2% ≥ 40 years 87.5%	NR	N	Memorial University
Edwards et al. [26]	USA and Poland	Patients	All had received a prescription for GLP‐1 RAs	Adults with T1DM ever treated with GLP‐1RA and/or SGLT2i for at least 90 days from two academic centres.	68	45 yrs, 73.5%	86.8% white, 8.8% black, 1.5% asian; 92.6% non‐hispanic, 5.9% hispanic	Y	No external funding
Ekenberg [27]	Sweden	Patients and HCPs	All patients were taking GLP‐1 RAs. HCPs prescribers of GLP‐1 RAs	Patients: Adults with T2DM and taking GLP‐1 RAs for at least 1 month HCPs: Experience of managing patients with T2DM and taking GLP‐1 RAs	7 patients & 3 HCPs (doctor, diabetes nurse, pharmacist)	Patients: 67.7 years, 71.4% HCPs: 44.3 years, 66.7%	NR	NR	NR
Flory et al. [28]	USA	HCPs	Experience of antidiabetic drugs (incl GLP‐1 RAs) for individuals with chronic kidney disease	Any general internist, family practitioner, nurse practitioner, physician's assistant, or endocrinologist was eligible.	2 endocrinologists, 6 general internists, 1 nurse	NR	NR	NR	Patient‐centred Outcomes Research Institute and NIH
Holmes‐Truscott et al. [29]	Australia	Patients	Current or previous use of GLP‐1 RAs	Adults who self‐reported a diagnosis of T2DM and that they had commenced (though not necessarily continued) GLP‐1 RA therapy in the past 3 years	19	(median) 64 years, 53%	NR	Y	Deakin University Deans Research Postdoctoral Fellowship; funding from the Australian Centre for Behaviour Research in Diabetes; Department of Health, Commonwealth Government of Australia
Kan [30]	Netherlands	Public	Have bought or thought about buying GLP‐1 RAs online to help with weight loss	Individuals, without medical prescriptions, who had purchased GLP‐1 RAs online in the past, or had not purchased online but were motivated to purchase online and had views and opinions about it	5	(range) 23–30 years, 80%	NR	NR	NR
Lamaro et al. [31]	Australia	Patients	Perspectives relating to GLP‐1 RA use as an adjunct to insulin therapy	Adults with a self‐reported T1DM diagnosis who had completed a quantitative online survey about treatment priorities	20	NR	NR	N	None
Lennon [32]	USA	Public social media posts	Personal experience of using GLP‐1 RA	TikTok videos identified through #Ozempic, with more than 10000 views, about personal experience of Ozempic/Semaglutide (videos of doctors explaining their medical opinion were excluded)	NR	NR	NR	NR	NR
Lindgreen et al. [33]	Denmark/UK	Patient/public social media posts	Experience of using GLP‐1 RAs in relation to binge eating	Postings and discussion about binge eating from a T2DM Facebook group over 8 months (October 2022–May 2023)	Group approx. 6500 members	53% > 55 years, 72%	NR	Y	Danish Diabetes Association; Novo Nordisk Foundation; NIHR
Matza et al. [16] and Boye et al. [15]	USA	Patients	Received tirzepatide for T2D treatment (trial)	Aged ≥ 18 years, insulin‐naive and T2DMinadequately controlled on stable treatment with metformin alone or in combination with an SGLT2 inhibitor.	28	57.6 years, 64.3%	Hispanic/Latino 32.1%, Not Hispanic/Latino 67.9%; Black/African American 25.0% Native Hawaiian/Pacific Islander 3.6%, White 57.1%, Multiple 3.6%, Other 10.7%	Y	Eli Lilly and Company
Paisey [34]	UK	Patients	Experience of taking GLP‐1RA	Purposive sampling of participants with T2DM with successful experience of incretin analogue therapy (GLP‐1 RAs)	15 (3 groups of 5)	NR	NR	NR	University of Plymouth
Polonsky et al. [35]	USA	Patients	Experience of taking GLP‐1RA	Adults with T2D, currently treated with a GLP‐1RA for at least 1 month at the time of screening or discontinued use within 1 year of screening but with a total of at least 1 month of treatment	16	58.3 years, 68.8%	White or Caucasian 81.2% Black or African American 12.5% Other 6.3%	Y	Novo Nordisk Foundation
Psarou [36]	UK	Patients	Experience of taking GLP‐1 RA (exenatide, liraglutide)	All patients with T2DM eligible for glucose‐lowering and/or antiobesity drug prescriptions registered with primary or secondary care services and required a medication change	24	61.6 years, 50%	NR	NR	Diabetes UK
Richards et al. [37]	UK	Patients	Experience of taking GLP‐1RA (semaglutide 0.5 mg)	Self‐paying consumers of Second Nature Health Habits Ltd intervention, between June 8, 2023, and July 22, 2023, and had completed 12 weeks. Eligible participants were adults with obesity, with access to and the ability to use a smartphone or tablet device.	113	46.7 years, 90.3%	NR	Y	NR
Ryden et al. [38]	Brazil, Germany, Japan and the UK	Patients & HCPs	Experience of taking GLP‐1 RA (exenatide once weekly, liraglutide once daily) or clinical expert	Adults currently taking GLP‐1 RAs and began taking the medication more than 2 months before screening or currently on an oral diabetes medication and without experience with GLP‐1 RA treatment for T2DM. HCPs were experts in drug development or diabetes/endocrinology	50 patients and 4 HCPs	Patients: 52.8 years, 46% HCPs: NR	NR	Y	AstraZeneca
Somani et al. [39]	USA	Public social media posts on Reddit	Any discussion relating to GLP‐1 RA	All GLP‐1 RA‐related discussions of all openly available Reddit content by searching for discussions containing the brand and generic names of available GLP‐1 RA drugs	391,461 unique discussions, including 71,982 posts and 319,479 comments from 116,216 unique authors	NR	NR	Y	NIH; American Heart Association; Doris Duke Foundation
Turner et al. [40]	UK	Patients	Experience of taking GLP‐1 RA	Aged 5–18 years with T2DM, overweight or obese and absence of pancreatic autoimmunity, secondary or monogenic diabetes	12	Range 14–18 (4 18–19 years); 50%	White British 67%, Asian 25%, Black Caribbean 8%	N	Diabetes UK

Abbreviations: GLP‐1 RA, Glucagon‐like peptide‐1 receptor agonists; HCP, healthcare professional; mg, milligrams; NIHR, National Institute of Health Research; NIH, National Institute of Health NR; not reported; SGLT2i, Sodium‐glucose co‐transporter‐2 inhibitors; T1DM, type 1 diabetes; T2DM, type 2 diabetes

**Table 2:** Summary of findings of included studies.

References	Study aim	Indication	Data collection setting	Data collection technique	Analysis approach	Summary of themes/findings
*Experiences of prescribing/deprescribing GLP‐1 RAs*						
Andreassen et al. [18]	To explore how Wegovy (semaglutide) is managed and negotiated in general practice in a Danish setting.	Weight loss	GP Practices	Ethnography, semi‐structured interviews and consultation observations	Discourse analysis	Four prominent discourses: a fundamental trust in the science; emphasis on health and individual responsibility in relation to weight loss; negotiating the cost of weight loss; patient‐led shared decision‐making
Bradley et al. [22]	Documenting the protocols of lifestyle medicine practitioners who engage in deprescribing medications after lifestyle interventions delivered with a goal of potential T2D remission.	T2DM	NR	Interviews	NR	Approaches to deprescribing; clinical team involved in deprescribing, prioritisation of which drugs to target first, aims of deprescribing, perceived positive and negative effects of medication deprescribing; factors considered when deprescribing (including adverse effects and costs); follow‐up processes
Ciemins et al. [24]	To understand the knowledge, attitudes, and beliefs of physicians and administrators in 4 US health systems about guidelines and the use of these new medication classes to inform the dissemination and translation of the guidelines into US HCOs	T2DM	Four healthcare organisations	Interview and survey	Grounded theory constant comparison approach to identify themes, guided by the CFIR domains	The two drug classes (GLP‐1 RAs and SGLT‐2i) were not broadly prescribed. Lack of knowledge of the guidelines; discomfort with their use; concerns with cost and insurance coverage. Treatments lacked technology support or physician or practice‐level reporting of prescribing patterns. No incentives for impacting quality metrics.
Flory et al. [28]	To better understand how providers think about the choice of antidiabetic drugs in T2DM with CKD, and to identify possible ways to improve decision‐making in this common, important clinical scenario	T2DM	Across New York State, mainly from academic medical practices in New York City.	Interviews Telephone or video conferencing	Analysis applied grounded theory to identify themes	Prescribing in T2D and CKD: metformin as first line drug; change in practices (rising interest in using newer medications over older; changes in guidelines); uncertainty; safety concerns; individuality of treatment. Defining and monitoring CKD and changing prescribing patterns with eGFR. Providers Uncertainty about guidelines for prescribing antidiabetic drugs in patients with T2DM and CKD emerged from providers.
*Experiences of taking GLP‐1 RAs for diabetes management*						
Ekenberg [27] **	To gain knowledge about how patients diagnosed with T2DM experience and understand their treatment with GLP‐1 RAs in Sweden.	T2DM	Two healthcare centres	Semi‐structured telephone interviews	Systemic text condensation ‐ thematic	Four main findings: (1) Patients were satisfied with treatment and preferred GLP‐1 RAs compared to their other treatments, regardless of level of effect. (2) GLP‐1 RAs may impact lifestyle by effecting appetite and hunger levels and by stabilising glucose levels. (3) The administration frequency was more important than route of administration. Preferred administration frequency impacted the opinions of GLP‐1 RAs and was influenced by how easy it was to remember taking the drug. (4) Patients generally had sufficient understanding of their treatment, but regular follow‐ups and extra explanations of decisions could increase treatment motivation and reduce worries. The healthcare staff confirmed most of the experiences collected from patients.
Holmes‐Truscott et al. [29]	To undertake an in‐depth exploration of the expectations and experiences of adults with T2DM regarding GLP‐1 RA use and (dis)continuation, as well as attitudes toward further injectable treatment intensification	T2DM	Nonclinical setting or telephone	Semi‐structured face‐to‐face or telephone interviews	Inductive template analysis	(1) Symbolism and stigma of injectable diabetes treatment; (2) administration and device preferences; (3) treatment convenience and social impact; (4) treatment efficacy and benefits; (5) negative treatment side‐effects. Some participants were more receptive to insulin therapy following GLP‐1RA use, others emphasised unique concerns about insulin
Lamaro et al. [31]	To investigate patient needs, preferences and priorities for T1DM management, and to assess, from the patient's perspective, the utility, suitability, and viability of GLP‐1 RAs as an adjunct to insulin therapy in T1DM	T1DM	Online (Teams)	Semi‐structured interviews	Thematic	Satisfaction with current treatment; Willingness to explore adjunct medications; Risk and side effects tolerance; Preferences for administrative route; Impact of non‐insulin adjuncts. Individuals on a noninsulin adjunct reported marked increases in satisfaction in weight, TDI, glycaemic stability, HbA1c and quality of life compared to when using insulin only
Matza et al. [16] Boye et al. [15]*	To better understand the impact of tirzepatide from the patients' point of view	T2DM	Six US clinical sites, located in California, Florida, North Carolina, Oklahoma and Texas, which had two sites	Telephone interviews	Content analysis	Perceived treatment benefits: Improved blood glucose/HbA1c (96%), Weight loss (93%), Decreased appetite (79%), Increased energy (79%), Improved sleep (29%), Improved blood pressure (21%), Psychological benefit (18%), Decreased nocturia (11%), Improved neuropathy (11%), Improved eyesight (7%), Increased ability to focus (7%). 93% of participants reported a positive impact on quality of life and daily activities. 64% reported a positive impact in daily activities, 64% reported increased ability to exercise during treatment, 43% reported positive impact on their ability to work. Everyone stated that the changes experienced during the trial were important. The most commonly reported improvements were: blood glucose, weight, appetite and energy. 96% reported that they would be willing to continue treatment with tirzepatide and 100% said they would recommend tirzepatide to others. Other themes: Injection device, dverse events
Paisey [34]	To explore the most appropriate dietary and lifestyle advice to enhance the efficacy and tolerability of incretin analogue treatment in T2DM, with a secondary question of ‘Can food choices explain why some patients with T2DM fail to respond to incretin analogue therapy?’	T2DM	Room in a hospital with audiovisual recording setup	Focus groups	Thematic analysis	Three broad themes emerged from analysis; the experience of ‘A Changed Relationship to Food and Eating’ set in context with links and interactions to both ‘The Medical Experience’ and ‘Social, Cultural and Emotional Influences’
Polonsky et al. [35]	To expand current understanding of patients' experiences, motivations and challenges relevant to their persistence with GLP‐1 RAtherapy	T2DM	Six clinical sites in the United States	Semi‐structured face‐to‐face interviews	Thematic analysis	The most common facilitators reflected perceptions that the treatment was contributing to tangible, positive results (primarily, glycaemic control and weight loss). Reduced or absent treatment burden was also highly valued. In general, both continuers and discontinuers broadly reported supportive experiences with their healthcare provider team when initiating a GLP‐1 RA. However, it appeared that continuers were more likely than discontinuers to receive clinically relevant information from their healthcare team, including facts about GLP‐1 RAs, likely treatment benefits, the importance of gradual dose titration, and the need to adjust diet after initiation, as well as ongoing support. The most common challenges leading to treatment discontinuation related to disappointment with the same two categories—in this case, lack of treatment efficacy and/or enhanced treatment burden (e.g., medication side effects or high costs)
Psarou [36]	To measure how expectations, beliefs and attitudes towards different diabetes treatments change over time focusing on medicines which cause weight loss, weight gain or are weight neutral	T2DM	Primary or secondary care	Semi‐structured face‐to‐face interviews	Framework	Emotions‐ Negative Feelings, My Diabetes is serious, My Diabetes is not serious, Expectations of Treatment (medicines), Expectations of Care, Negative Perceptions and Experiences with Medicines, Positive Perceptions and Experiences with Medicines, Self‐Regulation, Medicine Taking Behaviour, Strategies to overcome negative aspects of insulin, Lifestyle Behaviour
Ryden et al. [38]**	To inform attribute and attribute‐level selection to develop a discrete choice experiment survey designed to examine preferences for GLP‐1 RA treatments among patients with T2DM	T2DM	Telephone	Semi‐structured interview	Content and thematic analysis	Efficacy (82%), adverse effects (64%) and dosing frequency (58%) were important treatment‐related attributes. The most ideal device features were a small size/length of the pen (21%), ease of use (14%) and no preparation needed (14%) Factors important to doctors when choosing a treatment: hypoglycaemic events, glycated haemoglobin (HbA1c)/glucose levels, gastrointestinal/nausea issues, blood pressure, blood glucose monitoring, cost, number of injections per day.
Turner et al. [40]	To explore adolescents' views and experiences of different treatments for T2DM, to improve treatment concordance and consider how the current treatment pathway for adolescent T2DM could be improved.	T2DM	Telephone	Interviews	Thematic	Adolescents want treatments that are effective, discrete, easy to take and do not make them different from their peers. As liraglutide was described as effective, and surgery viewed as acceptable in certain circumstances, greater consideration should be given to their potential role in treating adolescent T2DM
*Experiences of taking GLP‐RAs for weight loss*						
Barnard‐Kelly et al. [21]**	To record the expectations and experiences of participants taking daily liraglutide injections during the pilot trial as well as the views of healthcare professionals about the feasibility of delivering the intervention in routine care	Weight loss	Community and in‐patient mental health centres	Semi‐structured face‐to‐face interviews	Content and thematic analysis	Key themes were despondency regarding prior medication associated weight gain, quality of life, impact of weight loss, practical aspects of participation including materials received and clinic attendance. Although some participants had reservations about the injections before the study, most of the trial completers reported no challenges in the timing of or administering the injections.
Donnan et al. [25]	To identify what factors influence patient preferences when considering a new medication for weight management	Weight loss	Zoom	Semi‐structured interviews and focus groups	Thematic analysis, inductive and deductive coding	(1) cost, (2) regimen, (3) side effects, (4) benefits and (5) non‐medication attributes. Cost of medications, lack of coverage by insurance companies, and stigma were identified as major barriers to accessing medications. There was agreement over the preference for a simple regimen, however there were diverse opinions on tolerability of side effects, desired benefits and route of administration. Themes where GLP‐1 RAs were specifically mentioned: cost and non‐medication attributes
Kan [30]	To explore the factors of online GLP‐1 RA purchasing behaviour, without medical prescription	Weight loss	Telephone	Semi‐structured in‐depth interviews	Thematic analysis	Multiple dominant specific beliefs, categorised under the COM‐B Model domains of Psychological Capability, Reflective Motivation and Social Opportunity. Participants were unconcerned about potential health and scam risks associated with online purchases. All participants showed optimism regarding the use and purchase of the prescription medicines. Awareness of medicine shortages emerged as a possible motivator to go online to obtaining GLP‐1 RAs
Richards et al. [37]	This service evaluation aimed to evaluate the preliminary impact of a remotely delivered semaglutide‐supported specialist weight management intervention for self‐paying adults living with obesity in the UK, including the feasibility and acceptability of this intervention	Weight loss	Online	Free text answers to questionnaire	Content analysis	Most participants had a positive or neutral experience of the intervention, with some reporting perceived benefits as early as 4 weeks (20%). However, benefits were only mentioned by a small number of participants at weeks 8 and 12 (3/78, 4% and 3/69, 4%, respectively).
*Experiences of taking GLP‐1 RAs for multiple indications*						
Edwards et al. [26]	To investigate the patient‐perceived reasons for initiation, perceived benefits, side effects experienced and willingness to continue or restart adjuvant treatment with GLP‐1 RA or SGLT2i in patients with T1DM	Multiple	Telephone in two academic clinical practices	Telephone survey	NR	Patients with T1DM report initiating adjuvant treatment with GLP‐1 RA and/or SGLT2i to improve glycaemic control and lose weight; most patients reported perceived benefits from these therapies. Side effects (including DKA) are reported more commonly in real life than in clinical trials.
*Social media‐based studies*						
Alvarez‐Mon et al. [17]	To investigate the content and key metrics of tweets referring to antiobesity drugs	Weight loss	Twitter Firehose data stream	Analysis of tweets	Thematic content analysis	Liraglutide and Semaglutide accumulated the most interest among Twitter users, highest frequency related to treatment efficacy. Tweets with a negative valuation of side effects were rarely in those mentioning liraglutide and semaglutide.
Arillotta et al. [19]	To assess the possible impact of GLP‐1 RAs on mental health in general as being perceived and discussed in popular open web platforms	Weight loss	Reddit, YouTube and TikTok posts/videos	Analysis of social media posts (AI used in analysis)	Content analysis	The most represented posts related to sleep‐related issues, including insomnia (*n* = 620 matches); anxiety (*n* = 353); depression (*n* = 204); and general mental health issues (*n* = 165). The Complex Interrelation between Weight and Overall Levels of Psychological Wellbeing; Weight Loss Medication Intake and Either Occurrence, or Improvement, of: Sleep Disturbances; Anxiety; ‘Food Noise’; Suicidal Ideation; Addictive Behaviour; Weight loss drugs; related safety and challenges in medications' access issues. Weight loss with GLP‐1 RAs was associated with either a marked improvement or, in some cases, a deterioration, in mood; increase/decrease in anxiety/insomnia; and better control of a range of addictive behaviours. Access to GLP‐1 RAs was a hot topic as well.
Arillotta et al. [20]	To explore the potential effects of GLP‐1 RAs on possible modifications relating to both drug/alcohol abuse intake and behavioural addictions	Weight loss	Reddit, YouTube and TikTok (though mainly Reddit)	Analysis of social media posts (AI used in analysis)	Thematic analysis	Five main themes: medication effects and side effects; lifestyle changes and weight management; substance use and cravings reduction; individual experiences and responses to GLP‐1 RAs; health risks, management and societal perceptions
Bremmer et al. [23]	To present the findings as a case example of the potential for social media data to inform questions concerning therapeutic efficacy and mechanisms in the context of drug repurposing	Any	Social media (Reddit)	Analysis of social media posts	Thematic analysis	Lost interest in alcohol; craving reduction; consumption patterns; satiety; subjective response; residual‐next day effects; participant characteristics; other.
Lennon [32]	To explore the discourse surrounding the off‐label use of Ozempic for weight loss on TikTok compared with WeightWatchers to expand the current understanding of how the medicalisation of obesity intersects with beauty ideals to incentive weight loss	Weight loss	TikTok	Analysis of TikTok videos/posts	Content analysis	Discussions about Ozempic resembled discussions about traditional diets, with some key differences. For example, the reliance on a medical framing of fatness to justify using medication for weight loss. People using Ozempic rely almost exclusively on the framing of obesity as a disease, illustrating how the use of medication for weight loss is only perceived as being acceptable if explicitly related to health, and not to vanity. The medicalisation of fatness plays a critical role in how people discuss Ozempic. One prominent theme was the use of biometrics and reporting details about the amount of weight lost while using the drug.
Lindgreen et al. [33]	To explore discussions about binge eating among members of a T2DM‐specific Facebook group	T2DM	Analysis of Facebook group discussion over 8 months	Analysis of social media posts	Interpretive description	One theme with two subthemes was developed from the data analysis. The theme ‘Sharing personal experiences and rectifying misconceptions’ elucidates how the Facebook group members interacted when discussing binge eating. The subthemes describe the topics that were discussed; binge eating triggers and inhibitors.
Somani et al. [39]	To characterise public perceptions about GLP‐1 RAs on Reddit	Any	Online, Reddit	Text scanning of Reddit posts	Topic modelling, using LLM and AI	168 topics and 33 groups focused on the GLP‐1 RA experience with weight loss, comparison of side effects between differing GLP‐1 RAs and alternate therapies, issues with GLP‐1 RA access and supply, and the positive psychological benefits of GLP‐1 RAs and associated weight loss. Notably, public sentiment in these discussions was mostly neutral‐to‐positive.

Abbreviations: AI, artificial intelligence; CFIR, consolidated framework for implementation research; CKD, chronic kidney disease; COM‐B, capability, opportunity, motivation ‐ behaviour model; CVD cardiovascular disease; eGFR, estimated glomerular filtration rate; GLP‐1 RA, glucagon‐like peptide‐1 receptor agonist; LLM, large language model; NR, not reported; SGLT2i, Sodium‐glucose co‐transporter‐2 inhibitors; TDI, total daily insulin; T2DM, type 2 diabetes mellitus; T1DM, type 1 diabetes mellitus;

* Linked papers

** Study also includes findings of the clinicians'/healthcare professionals' experiences of prescribing/deprescribing GLP‐1 RAs

### 3.3 Setting

Studies were predominantly conducted in high‐income Western countries. Eight were from the USA [16, 22, 23, 24, 28, 32, 35, 39], five from the UK [21, 34, 36, 37, 40], two from Italy [19, 20] and Australia [29, 31], and one each from Spain [17], Denmark [18], Canada [25], Sweden [27] and Netherlands [30]. Three studies were conducted in more than one country including USA and Poland [26], Denmark and the UK [33] and Brazil, Germany, Japan and the UK [38]. Studies were published from 2014 [34] to 2024 [18, 20, 23, 30, 31, 33, 39], although 85% of the studies were published within the last 3 years.

#### 3.3.1 Design

Studies can be grouped into those that directly spoke with patients/public (*n* = 11) [16, 25, 26, 29, 30, 31, 34, 35, 36, 37, 40], with healthcare professionals or clinicians (*n* = 4) [18, 22, 24, 28], those that were a mix of both (*n* = 3) [21, 27, 38], and those that were social media‐based studies (*n* = 7) [17] [19, 20, 23, 32, 33, 39]. The studies by Matza and colleagues were based on the same cohort of patients and therefore have been treated as one study [15, 16].

##### 3.3.1.1 Data Collection

Data was predominantly collected through interviews (*n* = 15), and where specified, these were either face‐to‐face (*n* = 4) [18, 21, 35, 36], telephone (*n* = 5) [16, 27, 30, 38, 40], online (*n* = 2) [25, 31] or a combination (*n* = 2) [28, 29]. In addition to interviews, Donnan and colleagues collected data through focus groups [25], Andreassen and colleagues undertook an ethnographic design also observing GP consultations [18], and Ciemins and colleagues incorporated a survey [24]. The study by Paisey and colleagues was the only study to solely use focus groups [34], whilst the study by Richards and colleagues used free text answers within a service evaluation questionnaire [37], and Edwards and colleagues used a telephone survey [26]. Whilst the setting of the studies in this group were often within a healthcare context (*n* = 10) [16, 18, 21, 24, 26, 28, 34, 35, 36, 37], it was often unclear which services the patients involved in these studies were based within or had access to. The data for social media‐based studies was derived from Twitter (*n* = 1) [17], Reddit (*n* = 4) [19, 20, 23, 39], Facebook (*n* = 3) [19, 20, 33] and TikTok (*n* = 3) [19, 20, 32].

##### 3.3.1.2 Data Analysis

Across all included studies, thematic analysis was the predominant method used for data analysis (*n* = 10) [17, 20, 23, 25, 27, 30, 31, 34, 35, 40], followed by content analysis (*n* = 4) [16, 19, 32, 37], or a combination of the two [21, 38]. Two studies applied a grounded theory approach to analysis [24, 28]. Other approaches for analysis within the studies exploring experiences of prescribing/deprescribing GLP‐1 RAs and taking GLP‐1 RAs for all indications, included discourse analysis [18], template analysis [29] and framework analysis [36]. Two studies did not describe the analysis process [22, 26]. For the social media‐based studies, Lindgreen and colleagues used an interpretive description [33], and Somani and colleagues used a large language model and artificial intelligence (AI) to create topic models from text scanning within the social media posts [39]. AI was also used in the analysis process in the study by Arillotta and colleagues [19, 20].

#### 3.3.2 Sample

##### 3.3.2.1 Patient/Public Studies

The sample size for all those studies that directly spoke with patients/public (*n* = 14), including those that were a mix of patients and healthcare professionals, ranged from five [30] to 113 [37]. Of these, 12 studies presented a mean/median age of the sample which ranged from 42.6 [21] to 67.7 years [27]. The studies with the two youngest cohorts provided age ranges of their sample; for Kan and colleagues, this was 23–30 years [30], and Turner and colleagues 14–18 years [40]. Donnan and colleagues reported that 76.2% of their sample was ≥ 40 years [25]. The percentage of females was reported in 12 studies [16, 21, 25, 26, 27, 29, 30, 35, 36, 37, 38, 40] and this ranged from 30.8% [21] to 90.3% [37]. Racial characteristics of the sample were only reported in five studies [16, 21, 26, 35, 40] and in each case the sample was predominantly white/Caucasian ranging from 57.1% [16] to 88% (specifically white British and Irish) [21]. Of the two studies that reported ethnicity, the sample was predominantly non‐Hispanic/Latino [16, 26].

Nine studies specifically listed adults (or age ≥ 18 years) within their inclusion criteria [16, 25, 26, 27, 29, 31, 35, 37, 38], with the study by Barnard–Kelly and colleagues narrowing this to patients aged between 21 and 64 years [21]. One study targeted a younger cohort, aged between 5 and 18 years [40]. Eight studies specifically included patients with T2DM [16, 27, 29, 34, 35, 36, 38], two type 1 diabetes mellitus (T1DM) [26, 31], two obesity [25, 37] and one included patients with T2DM who were overweight/obese [40]. The study by Barnard‐Kelly and colleagues included overweight/obese patients with severe mental illness [21].

##### 3.3.2.2 Clinician/Healthcare Professionals

Of the studies that included clinicians or healthcare professionals (*n* = 7) sample size ranged from 2 [21] to 20 [24]. The specific roles of health professionals were mentioned in three studies [18, 28]. Collectively these included seven general practitioners, six general internists, six nurses, two endocrinologists, one junior doctor and one pharmacist. Only one study mentioned age and percentage of females within their sample of healthcare professionals, this being 44.3 years and 66.7% [27] respectively.

##### 3.3.2.3 Social Media‐Based Studies

The data source of the social media‐based studies (*n* = 7) included analysis of tweets (*n* = 1) [17], Reddit posts (*n* = 4) [19, 20, 23, 39], YouTube (*n* = 2) [19, 20], TikTok videos (*n* = 3) [19, 20, 32] and Facebook group postings (*n* = 1) [33]. The samples ranged from 1557 posts [23] to 780,382 tweets [17]. Only the study by Lindgreen and colleagues reported the age and percentage of females within the sample, with 55% being > 55 years old and 75% female [33].

#### 3.3.3 Phenomenon of Interest

**Figure 2:**
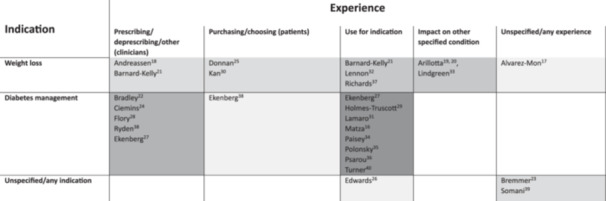
Overview of the focus of included studies. Darker shades represent cells with more studies, lighter shades represent cells with fewer studies.

There was a range of experiences relating to GLP‐1 RAs, as summarised in Figure 2.

Out of the seven studies interviewing clinicians or healthcare professionals, six were speaking to them with respect to prescribing or deprescribing. Out of these six, only the study by Andreassen and colleagues focused on GLP‐1 RAs for weight loss, and in this study, the primary care providers discussed the use of Wegovy (semaglutide) [18]. Just one study focused on deprescribing and that was the study by Bradley and colleagues who were interested in using GLP‐1 RAs to manage T2DM [22]. Four studies captured the thoughts of healthcare practitioners/clinical experts about prescribing GLP‐1 RAs in the context of T2DM [24, 27, 28, 38], one specifically in patients with chronic kidney disease [28]. The one study not about prescribing or deprescribing was the study by Bernard‐Kelly and colleagues, where healthcare professionals were interviewed about the logistics and recruitment of the pilot study involving liraglutide for weight loss in patients with severe mental illness [21].

Of the 14 studies that directly spoke with patients/public, the experiences of three studies were derived from either the purchasing or choosing of GLP‐1 RAs for weight management [25, 30], or diabetes management [38]. In eight studies, the experiences involved the taking of GLP‐1 RAs with either a current or previous prescription, six of these as a T2DM treatment [27, 29, 34, 35, 36, 40], one as an adjunct to insulin for T1DM [31], and one for multiple indications [26]. In two studies, experiences with GLP‐1 RAs were as part of a clinical trial and here specific GLP‐1 RAs were reported, one using liraglutide for weight loss patients with severe mental illness [21], and one tirzepatide for T2DM [16]. The experience with GLP‐1 RAs in the remaining study in this group was specifically with semaglutide as part of a semaglutide‐supported specialist weight management intervention for fee‐paying adults [37].

In two of the social media‐based studies, discussions were not specifically related to the use of the drugs but any discussion that included GLP‐1 RAs [17, 39]. The experiences of five studies in this category related explicitly to the use of the drugs [19, 20, 23, 32, 33] and more specifically, one was in relation to binge eating [33], one to mental health issues [19], one substance use/addiction [20], and one to alcohol use with GLP‐1 RAs [23]. Five studies included data related to specific GLP‐1 RAs, these being solely semaglutide [32], liraglutide and/or semaglutide [17], and semaglutide and/or tirzepatide [19, 20, 23].

#### 3.3.4 Funding/Conflict of Interest

Fifteen of the 25 studies reported on conflict of interest [16, 19, 20, 22, 24, 25, 26, 29, 31, 33, 35, 37, 38, 39, 40] and out of these eleven declared some form of conflict [16, 19, 20, 24, 26, 29, 33, 35, 37, 38, 39]. Details of funding sources were reported in 20 studies [16, 17, 18, 19, 20, 21, 22, 24, 25, 26, 28, 29, 31, 33, 34, 35, 36, 38, 39, 40] and seven of these were partially or fully funded by a pharmaceutical company [16, 18, 21, 24, 33, 35, 38]. Ten studies did not include any conflict‐of‐interest statement [15, 16, 19, 21, 25, 26, 28, 30, 32, 34].

#### 3.3.5 Overview of Findings

Table 2 displays the key findings of the included studies. The studies are presented according to the five key areas addressed by the papers: experiences of prescribing/deprescribing GLP‐1 RAs, experiences of patients/public purchasing or choosing GLP‐1 RAs, social media‐based studies, and experiences of carers and GLP‐1 RAs.

##### 3.3.5.1 Experiences of Prescribing/Deprescribing GLP‐1RAs

Only seven of the 24 studies included experiences of clinician/healthcare professionals prescribing/deprescribing GLP‐1 RAs [18, 21, 22, 24, 27, 28, 38]. The bulk of these (*n* = 5) related to GLP‐1 RAs as a diabetic treatment option [22, 24, 27, 28, 38]. Three studies had a higher focus on GLP‐1 RAs [18, 21, 38], the other four discussed GLP‐1 RAs alongside several other medications [22, 24, 27, 28]. Only two related to prescribing GLP‐1 RAs specifically for weight‐loss purposes [18, 21]. In general, these seven studies highlighted some of the complexities, practicalities and costs involved in prescribing GLP‐1 RAs and the considerations for adverse events of taking GLP‐1 RAs. Where the studies by Ciemins and Flory and colleagues reported some uncertainties around the use of newer drugs including GLP‐1 RAs [24, 28], Andreassen and colleagues reported a general willingness and trust in the medication and familiarity with its previous use as a diabetic medication [18]. Whilst there were only a few studies in this group, it is worth highlighting that perhaps the richest qualitative evidence of all included studies was from the ethnographic study by Andreassen and colleagues which included interviews from 11 healthcare professionals across three GP practices and observations of 273 consultations, 27 of which involved discussions around the use of Wegovy (semaglutide) for weight loss purposes [18].

##### 3.3.5.2 Experiences of Patients/Public Purchasing or Choosing GLP‐1 RAs

Three studies looked at the experiences of patients/public choosing or purchasing GLP‐1 RAs [25, 30, 38]. Two of these discussed factors, thoughts and preferences to identify reasons for choosing GLP‐1 RAs as a medication when provided with several options, one with regard to diabetes management [38], and one weight‐loss (this being one of only two studies to use focus groups as a data collection method) [25]. One focused on the purchasing of GLP‐1 RAs for weight loss without a medical prescription [30]. These studies had a greater focus on GLP‐1 RAs generally although experiences were not solely related to taking the medication.

##### 3.3.5.3 Experiences of Patients/Public Taking GLP‐1 RAs

Out of the 12 studies that elicited patient experiences of taking GLP‐1 RAs, only three were based on GLP‐1 RAs for weight loss [21, 32, 37]. These focused on GLP‐1 RAs, liraglutide and semaglutide specifically, however one was in the context of a pilot trial [21], one used content analysis to evaluate a remotely delivered semaglutide‐based weight management programme [37], and one was a social media analysis of off‐label use [32].

Patient experience of using GLP‐1 RAs for diabetes management accounted for the largest group of studies (*n* = 8) [16, 27, 29, 31, 34, 35, 36, 40], and generally, there was a higher focus on GLP‐1 RAs. Seven of these studies used interview as the data collection method [16, 27, 29, 31, 34, 35, 36, 40], one used a focus group [32], and analysis primarily was using thematic techniques (*n* = 5) [25, 29, 32, 33, 38]. Studies were carried out within a healthcare context, although the services that patients were seen within was generally not clear. Several of the summary findings did report some positive changes and satisfaction with GLP‐1 RAs as a treatment option when compared to other medications [16, 27, 31, 35]. Common themes were identified around treatment practicalities, the want for an easy simple regime, routes of administration, treatment adherence, side effects and social and emotional factors. The impact on weight whilst taking these drugs for diabetic management was a key concept identified in six studies [16, 27, 31, 35, 36, 40]. Two studies highlighted follow‐up and ongoing support and explanations with healthcare providers about clinically relevant information that would aid decision‐making, as potentially important factors for increased motivation and continuation of treatment [27, 35].

One study investigated reasons for initiating or discontinuing GLP‐1 RAs and whilst the population were diabetic, considerations for use included both weight loss and glycaemic control. This telephone‐based survey did not describe analysis methods and GLP‐1 RAs were not discussed in isolation [26].

##### 3.3.5.4 Social Media‐Based Studies

Studies that used social media content generally used descriptive methods of analysis. Four focused on weight loss [17, 19, 20, 32], one on diabetes management [33], and one on any indication [23, 39]. These studies highlight strong public interest in GLP‐1 RAs and raise discussion points around public perceptions or experiences of use, however, the origin of social media posts and therefore the context of drug use was unclear.

##### 3.3.5.5 Experiences of Carers and GLP‐1 RAs

There was no evidence about the views, experiences or perceptions of carers regarding the use of GLP‐1 RAs.

## 4 Discussion

This is the first scoping review to capture the state of evidence relating to the experiences, views and perceptions of patients, carers and clinicians of GLP‐1 RAs for any indication. This review was important because, despite the abundance of clinical effectiveness trials on GLP‐1 RAs, it clearly shows that the evidence about experiences of these drugs is lagging behind. This was an issue highlighted by our PPIE group, who asked questions about the reality of using GLP‐1 RAs, beyond effect sizes for weight loss or safety risks. Such experience data, from patients, carers and clinicians, may be leveraged to help the development of medicines, or to support their delivery, and as such form a vital component of the broader picture of effectiveness and acceptability of a drug [41].

We conducted a thorough search for relevant literature through bibliographic databases, trial registries, citation chasing and hand searching of relevant systematic reviews. We identified 25 studies (from 26 reports) that involved qualitative methods and included experiences of GLP‐1 RAs. Despite this number of studies, they were spread across all possible viewpoints and indications, so we found that there was little rich evidence to serve the range of patients, carers and prescribers who may be interested in the use of GLP‐1 RAs. Across all groups of studies, there were limitations in terms of how focused studies were on GLP‐1 RAs and the depth or quality of evidence available, especially with regard to the studies related to weight loss.

Only seven studies involved interactions with healthcare professionals/clinicians and out of these only two were focused on weight loss. GLP‐1 RAs have recently gained a large amount of media coverage with regard to weight loss and as highlighted by the large number of discussions across various social media platforms in the social media‐based studies in this review, these drugs are of high interest within the public domain. It can be assumed therefore that patients/public will approach their healthcare providers with questions about these medications. The importance of these discussions has been documented [42], as have the complexities involved with discussing weight with patients with overweight and obesity [43]. It is imperative that clinicians and healthcare professionals are equipped to have informed and engaged conversations with their patients, and carers of these patients, to not only aid the decision‐making process but also to offer ongoing support for those that take these medications. What we don't know, however, because the qualitative evidence is lacking, is what clinicians and healthcare providers want to know about these medications, what support they might need to feel confident prescribing or deprescribing these drugs for weight loss and what their views might be on service delivery and wrap‐around care that might be needed with these medications.

The greatest section of coverage provided by the evidence in this review was the patient experience of taking GLP‐1 RAs for the management of diabetes (eight studies). Only three studies provided the patient experience of taking GLP‐1 RAs for weight loss and these were limited by quantity and depth of data analysis and focus of phenomenon of interest. This collection of studies leaves an array of gaps in understanding of the patient experience of the range of weight loss drugs available. It fails to inform us of the support patients might need when taking GLP‐1 RAs for weight loss. This body of evidence is vital when considering development of services linked to these medications especially when there is research highlighting that including user perspectives into health service development can enhance care delivery [44]. Given that there have been over 28 network meta‐analyses of the effectiveness of GLP‐1 RAs for weight loss since 2020, for there to only be a handful of qualitative studies on the same drugs for the same purpose, highlights just how far behind qualitative evidence is and this needs to be addressed [6]. Whilst some of the effectiveness studies on GLP‐1 RAs collect patient‐reported outcomes such as health‐related quality of life, this type of data does not provide us with the same quality of insight into experiences as qualitative research.

None of the 25 included studies captured the experiences, views or perceptions of GLP‐1 RAs from a carer perspective. This highlights a gap in the evidence which needs to be addressed. According to the 2021 census, there are an estimated 5.7 million unpaid carers in the UK [45]. Part of the NHS long term plan has been to shift more care out of the hospital setting and into the community [46], the role of the caregiver in supporting those with complex health conditions, like obesity, is perhaps therefore even more involved, especially with increased remote management in conditions like diabetes [47]. The views and experiences of carers with regard to medications such as GLP‐1 RAs must be sought to understand complexities and highlight support needs from this perspective.

Eleven of the 15 studies that provided a conflict‐of‐interest statement reported some form of conflict, and seven studies were partially or fully funded by pharmaceutical companies. Ten studies did not provide any conflict‐of‐interest statement. Full disclosure of conflicts of interest is important for improving transparency within research and not all conflicts of interest is negative [48]. However, it is unclear whether the inclusion of a conflict‐of‐interest statement is sufficient to give confidence in transparency [49], and from the information provided in the included studies, it is not possible to fully interpret the impact commercial interests have had on the findings of the research. The lack of transparency about the links between the pharmaceutical companies and the research adds further limitations to this body of evidence and threatens the public's trust in the findings, a point highlighted by our PPIE group.

This review highlighted that whilst there is some qualitative evidence related to the experiences, views and perceptions of patients and clinicians of GLP‐1 RAs, this was mostly with respect to diabetes management and mostly from a patient perspective. Where authors detailed the specific GLP‐1 RAs that were being used, a range of drugs and doses were captured, including semaglutide [18, 37], liraglutide [21, 36], tirzepatide [16] and exenatide [36, 38]. However, based on the publication dates of studies, and the stated indications for the drugs, more recent doses of tirzepatide and semaglutide intended for weight loss were likely infrequently targeted. Perceptions of these drugs may have been captured within the studies that analysed social media posts (e.g. Arillotta et al. [19, 20]); however, the limitation with this data source means this level of detail was not available for these studies. Whilst the experiences of taking tirzepatide for diabetes were captured in exit interviews from the trial by Matza and colleagues, the content analysis techniques used, and descriptive results limit the degree of interpretation that could be gained [16].

PERSPEX members were disappointed with the poor quality of the evidence in this review which they felt did not reflect the importance of the topic to patients. Future collaborations with PERSPEX will enable us to develop a dissemination strategy of benefit to patients and carers.

### 4.1 Future Research

There is an urgent need for high‐quality qualitative evidence to explore the experiences of patients, carers and clinicians with respect to the use of GLP‐1 RAs, particularly those indicated for weight loss. This type of qualitative evidence is crucial to facilitate decision‐making, understand patients', carers' and clinicians' support needs, and inform service development. Qualitative evaluations could be conducted alongside new randomised controlled trials, or as stand‐alone studies seeking to recruit a diverse range of participants. Qualitative methods that elicit rich experience data would be particularly valuable.

Once a body of primary evidence is established, qualitative evidence synthesis is required to inform commissioning/clinical practice/government guidelines. Until a more complete understanding of patient, carer and clinician experiences' is obtained, some caution should be applied to the commissioning of services supporting the delivery of GLP‐1 RAs.

### 4.2 Limitations and Strengths

This review was limited by the quantity and quality of primary qualitative research exploring patient, clinician and especially carer experiences of using of GLP‐1 RAs for any indication and but especially for weight loss. There were limitations with data collection and data analysis techniques in the included studies, with often a limited focus on GLP‐1 RAs, reducing the value of in‐depth interpretation of findings. The lack of transparency around the potential impact of conflicts of interest on the findings is a further limitation with this body of evidence.

Our scoping review is underpinned by a thorough search strategy encompassing database searching and supplementary search methods (including citation chasing and searching of Google Scholar and the pre‐print database MedRxiv). The search summary table in Appendix B highlights that unique papers were only found in Medline and Google Scholar. We kept our inclusion criteria of population broad enough to ensure we captured all possibly relevant studies, and we included GLP‐1 RAs for any indication to widen the reach of evidence that might be available.

## 5 Conclusions

Whilst this scoping review did identify primary evidence involving qualitative methods and including experiences and views of patients and clinicians in the use of GLP‐1 RAs, significant gaps in this evidence were identified, in particular relating to the use of these drugs for weight loss purposes. No evidence was found that captured the experiences, views and perceptions of carers and GLP‐1 RAs across any indication. These gaps need to be addressed by conducting more qualitative research, using methods that generate rich data that capture a diversity of real‐life experiences from patients, carers and clinicians so that we can better understand these drugs beyond just evaluating their effectiveness.

## Author Contributions

**Sam Febrey:** conceptualisation, investigation, methodology, project administration, visualisation, writing – original draft preparation, writing – review and editing. **Michael Nunns:** conceptualisation, investigation, methodology, project administration, supervision, visualisation, writing – original draft preparation, writing – review and editing. **Jill Buckland:** conceptualisation, investigation, methodology, visualisation, writing – original draft preparation, writing – review and editing. **Rebecca Abbott:** conceptualisation, investigation, methodology, visualisation, writing‐review and editing. **Alison Bethel:** conceptualisation, investigation, methodology, visualisation, writing‐original draft preparation, writing‐review and editing. **Rebecca Whear:** conceptualisation, methodology, writing – review and editing. **Kate Boddy:** conceptualisation, methodology, writing‐original draft preparation. **G. J. Melendez‐Torres:** funding acquisition, conceptualisation, methodology, writing‐review and editing. **Jo Thompson Coon:** funding acquisition, conceptualisation, methodology, writing‐review and editing. **Liz Shaw:** conceptualisation, investigation, methodology, project administration, supervision, writing‐original draft preparation, writing‐review and editing.

## Ethics Statement

The authors have nothing to report.

## Conflicts of Interest

The authors declare no conflicts of interest.

## Rights Retention

For the purpose of open access, the author has applied a Creative Commons Attribution (CC BY) licence to any Author Accepted Manuscript version arising from this submission.

## Information Governance Statement

During the conduct of this report, we were not required to handle any personal information.

## Supporting information

Supplementary Information

Supplementary Information

Supplementary Information

## Data Availability

The data that support the findings of this study are available from the corresponding author upon reasonable request.
