# Supplementary material for: What Are the Experiences, Views and Perceptions of Patients, Carers and Clinicians of Glucagon‐like Peptide‐1 Receptor Agonists (GLP‐1 RAs)? A Scoping Review
Source: Health Expect. 2025 Apr 14;28(2):e70251. doi: 10.1111/hex.70251 (PMC11995417; doi:10.1111/hex.70251)
Supplement: Supplementary file 1 — Supplementary Information [file HEX-28-e70251-s003.docx]

## Appendix A: Search strategies

Ovid MEDLINE(R) ALL <1946 to June 21, 2024>

1 Semaglutide*.tw.

2 Ozempic*.tw.

3 Rybelsus*.tw.

4 Wegovy*.tw.

5 Liraglutide*.tw.

6 Victoza*.tw.

7 Saxenda*.tw.

8 Tirzepatide*.tw.

9 Mounjaro*.tw.

10 Exenatide*.tw.

11 Byetta*.tw.

12 Dulaglutide*.tw.

13 Trulicity*.tw.

14 Lixisenatide*.tw.

15 Lyxumia*.tw.

16 or/1-15

17 glucagon like peptid* one.tw.

18 "glucagon like peptid* 1".tw.

19 *Glucagon-Like Peptides/

20 exp Glucagon-Like Peptide 1/

21 "glp 1*".tw.

22 glp-1*.tw.

23 or/16-22

24 interview*.tw.

25 qualitative*.tw.

26 experienc*.tw.

27 exp Qualitative Research/

28 (perception* or perceiv*).tw.

29 Focus Groups/

30 "focus group*".ti,ab.

31 thematic*.tw.

32 24 or 25 or 26 or 27 or 28 or 29 or 30

33 23 and 32

**APA PsycInfo** <1806 to June Week 2 2024>

1 Semaglutide*.tw.

2 Ozempic*.tw.

3 Rybelsus*.tw.

4 Wegovy*.tw.

5 Liraglutide*.tw.

6 Victoza*.tw.

7 Saxenda*.tw.

8 Tirzepatide*.tw.

9 Mounjaro*.tw.

10 Exenatide*.tw.

11 Byetta*.tw.

12 Dulaglutide*.tw.

13 Trulicity*.tw.

14 Lixisenatide*.tw.

15 Lyxumia*.tw.

16 or/1-15

17 glucagon like peptid* one.tw.

18 "glucagon like peptid* 1".tw.

19 *Glucagon-Like Peptides/

20 "glp 1*".tw.

21 glp-1*.tw.

22 exp Qualitative Methods/

23 interview*.tw.

24 (("semi-structured" or semistructured or unstructured or informal or "in-depth" or indepth or "face-to-face" or structured or guide or guides) adj3 (discussion* or questionnaire*)).ti,ab.

25 (focus group* or qualitative or ethnograph* or fieldwork or "field work" or "key informant").ti,ab,id.

26 qualitative study.md.

27 exp qualitative research/

28 exp Interviews/

29 exp Group Discussion/

30 22 or 23 or 24 or 25 or 26 or 27 or 28 or 29

31 16 or 17 or 18 or 19 or 20 or 21

32 30 and 31

**Cinahl Ultimate Ebscohost**

| Run 20 June 2024  **#** | **Query** |
| --- | --- |
| S1 | TI semaglutide* OR AB semaglutide* |
| S2 | TI Ozempic* OR AB Ozempic* |
| S3 | TI Rybelsus* OR AB Rybelsus* |
| S4 | TI Wegovy* OR AB Wegovy* |
| S5 | TI liraglutide* OR AB liraglutide* |
| S6 | TI Victoza* OR AB Victoza* |
| S7 | TI Saxenda* OR AB Saxenda* |
| S8 | TI tirzepatide* OR AB tirzepatide* |
| S9 | TI Mounjaro* OR AB Mounjaro* |
| S10 | TI exenatide* OR AB exenatide* |
| S11 | TI Byetta* OR AB Byetta* |
| S12 | TI dulaglutide* OR AB dulaglutide* |
| S13 | TI Trulicity* OR AB Trulicity* |
| S14 | TI lixisenatide* OR AB lixisenatide* |
| S15 | TI Lyxumia* OR AB Lyxumia* |
| S16 | (MH "Glucagon-Like Peptide-1 Receptor Agonists") |
| S17 | TI "Glucagon-Like Peptide-1 Receptor Agonist*" OR AB "Glucagon-Like Peptide-1 Receptor Agonist*" |
| S18 | TI GLP-1 OR AB GLP-1 |
| S19 | TI glucagon like peptid* one OR AB glucagon like peptid* one |
| S20 | TI "glucagon like peptid* 1" OR AB "glucagon like peptid* 1" |
| S21 | (S1 OR S2 OR S3 OR S4 OR S5 OR S6 OR S7 OR S8 OR S9 OR S10 OR S11 OR S12 OR S13 OR S14 OR S15 OR S16 OR S17 OR S18 OR S19 OR S20) |
| S22 | (MH "Qualitative Studies+") |
| S23 | (TI qualitative* OR AB qualitative* OR TI interview* OR AB Interview* OR TI experienc* OR AB experienc*) |
| S24 | TI ( view* or perspectiv* ) OR AB ( view* or perspectiv* ) |
| S25 | TI focus group* OR AB focus group* |
| S26 | (MH "Focus Groups") |
| S27 | S22 OR S23 OR S24 OR S25 OR S26 |
| S28 | S21 AND S27 |

**Google scholar**

- Experiences taking semaglutide qualitative to page 6
- Experiences taking liraglutide qualitative to page 6
- Experiences taking tirzepatide qualitative to page 4
- Experiences taking GLP-1 receptor agonists qualitative to page 4
- Perceptions taking GLP-1 receptor agonists qualitative to page 4

**MedRxiv** “GLP-1 and qualitative”

**Proquest Dissertations & Theses**

(noft(semaglutide OR wegovy OR ozempic OR rybelsus OR liraglutide OR victoza OR saxenda OR tirzepatide OR mounjaro OR exenatide OR byetta OR dulaglutide OR trulicity OR lixisenatide OR Lyxumia OR "glucagon like peptid* 1" OR GLP-1 OR "GLP 1") OR subject.Exact("Glucagon like peptide-1" OR "Glucagon like peptide 1")) AND (noft(qualitative OR interview* OR experienc* OR perspectiv* OR thematic*) OR subject(qualitative research))
