# Supplementary material for: What Are the Experiences, Views and Perceptions of Patients, Carers and Clinicians of Glucagon‐like Peptide‐1 Receptor Agonists (GLP‐1 RAs)? A Scoping Review
Source: Health Expect. 2025 Apr 14;28(2):e70251. doi: 10.1111/hex.70251 (PMC11995417; doi:10.1111/hex.70251)
Supplement: Supplementary file 2 — Supplementary Information [file HEX-28-e70251-s001.docx]

# Appendix B: Search summary table
