# Supplementary material for: What Are the Experiences, Views and Perceptions of Patients, Carers and Clinicians of Glucagon‐like Peptide‐1 Receptor Agonists (GLP‐1 RAs)? A Scoping Review
Source: Health Expect. 2025 Apr 14;28(2):e70251. doi: 10.1111/hex.70251 (PMC11995417; doi:10.1111/hex.70251)
Supplement: Supplementary file 3 — Supplementary Information [file HEX-28-e70251-s002.docx]

# Appendix C: Reasons for study exclusion at full text

**Reasons for study exclusion**

| **Author, Year** | **Title** with hyperlink where available | **Reason for exclusion** |
| --- | --- | --- |
|  | **Duplicate records n=2** |  |
| Holmes-Truscott, 2022 | 'For me, it didn't seem as drastic a step as being controlled by insulin': A qualitative investigation of expectations and experiences of non-insulin injectable therapy among adults with type 2 diabetes <https://doi.org/10.1111/dme.14681> | Duplicate record |
| Thom, 2020 | Weight loss maintenance: physiological, psychological and clinical perspectives | Duplicate record |
|  | **No Full text n=2** |  |
| 2022 | Effect of Glucagon-like Peptide 1 (GLP1) Receptor Agonists on Mental Health in Patients With Obesity and Type 2 Diabetes (T2D): a Mixed Methods Study | No full text |
| Collins, 2024 | # 1706263 Qualitative Insights on Meaningful Change in Physical Functioning in SURMOUNT-4 Participants | No full text |
|  | **Phenomenon of interest n=10** |  |
| Berry-Price, 2024 | Exploring the Lived Experience of Self-Care in Young Adults with Type 2 Diabetes | Phenomenon of interest |
| Brown, 2021 | Health care providers’ emotional responses to their patients’ hypoglycemic events: qualitative findings from the Inhypo-DM study, Canada | Phenomenon of interest |
| Fujioka, 2015 | Usability of the Novel Liraglutide 3.0 mg Pen Injector Among Overweight or Obese Adult Patients With or Without Prior Injection Experience <https://dx.doi.org/10.1177/1932296815593295> | Phenomenon of interest |
| Gauthier, 2014 | Perspectives and experiences of health care professionals and patients regarding treatments for type 2 diabetes | Phenomenon of interest |
| Khatib, 2022 | Perceptions of injectable therapies with cardiovascular benefit: an ACNAP survey of healthcare professionals to explore facilitators and barriers <https://dx.doi.org/10.1093/eurjcn/zvab106> | Phenomenon of interest |
| Levkovich, 2021 | Perceptions among diabetic patients in the ultra-orthodox Jewish community regarding medication adherence: a qualitative study <https://doi.org/10.1186/s12889-021-11619-6> | Phenomenon of interest |
| Roborel de Climens, 2020 | Understanding Reasons for Treatment Discontinuation, Attitudes and Education Needs Among People Who Discontinue Type 2 Diabetes Treatment: Results from an Online Patient Survey in the USA and UK <https://dx.doi.org/10.1007/s13300-020-00843-9> | Phenomenon of interest |
| Salvia, 2022 | Managing type 2 diabetes or prediabetes and binge eating disorder: a qualitative study of patients' perceptions and lived experiences 10.1186/s40337-022-00666-y | Phenomenon of interest |
| Samuels, 2024 | Patients experience with preoperative use of anti-obesity medications and associations with bariatric surgery expectations <https://doi.org/10.1101/2024.04.14.24305798> | Phenomenon of interest |
| Williamson, 2014 | Health care provider management of patients with type 2 diabetes mellitus: analysis of trends in attitudes and practices <https://dx.doi.org/10.3810/pgm.2014.05.2764> | Phenomenon of interest |
|  | **Population n=5** |  |
| Boye, 2019 | Patient preferences and health state utilities associated with dulaglutide and semaglutide injection devices among patients with type 2 diabetes in Italy <https://dx.doi.org/10.1080/13696998.2019.1609482> | Population |
| Milder, 2021 | Prescribing of SGLT2 inhibitors in primary care: a qualitative study of general practitioners and endocrinologists | Population |
| Mostafavi, 2021 | The psychosocial barriers to medication adherence of patients with type 2 diabetes: a qualitative study | Population |
| Spreckley, 2022 | Patient insights into the experience of trying to achieve weight-loss and future expectations upon commencement of a primary care-led weight management intervention: A qualitative, baseline exploration | Population |
| Stauder, 2014 | Comparative Assessment of Lixisenatide, Exenatide, and Liraglutide Pen Devices: A Pilot User-Based Study <https://dx.doi.org/10.1177/1932296813511733> | Population |
|  | **Publication status n=6** |  |
| Flemming, 2023 | Pediatric diabetes: an exploration of shared care in Nova Scotia | Publication status |
| Keating, 2023 | Semaglutide and social media: implications for young women with polycystic ovarian syndrome | Publication status |
| Kufahl, 2024 | Prescribing GLP-1s for Obesity in Primary Care | Publication status |
| Qin, 2016 | GLP-1 Receptor Agonist Treatment-Related Features Important to Type 2 Diabetes Mellitus (T2DM) Patients: A Preference Study in Germany | Publication status |
| Strizek, 2019 | PDB112 EXPERIENCES OF PATIENTS WITH TYPE 2 DIABETES MELLITUS PATIENTS IN THE KINGDOM OF SAUDI ARABIA | Publication status |
| Thom, 2020 | Weight Loss Maintenance: Physiological, Psychological and Clinical Perspectives | Publication status |
|  | **Study design** |  |
| Al Hayek, 2022 | Evaluation of Patient-Reported Satisfaction and Clinical Efficacy of Once-Weekly Semaglutide in Patients with Type 2 Diabetes: An Ambispective Study <https://dx.doi.org/10.1007/s12325-022-02053-0> | Study Design |
| Albargawi, 2024 | Efficacy and Safety of Injectable Dulaglutide 1.5 mg Among Type 2 Diabetes Patients in Clinics at King Saud Medical City, Riyadh, Saudi Arabia  <https://dx.doi.org/10.1007/s44197-024-00207-7> | Study Design |
| Banjara, 2021 | Patients’ Preferences for Second-Line Pharmacological Agents in Type 2 Diabetes | Study Design |
| Boye, 2021 | Patient Perceptions of and Preferences Between Characteristics of Injectable Diabetes Treatments  <https://dx.doi.org/10.1007/s13300-021-01097-9> | Study Design |
| Chen, 2020 | The Unmet Medical Needs of Current Injectable Antidiabetic Therapies in China: Patient and Health Care Professional Perspectives <https://dx.doi.org/10.1016/j.clinthera.2020.06.006> | Study Design |
| Christensen, 2013 | Detection of adverse drug reactions among ordinary users of liraglutide on the occasion of drug dispensing in the community pharmacy setting  <https://dx.doi.org/10.1097/PTS.0000000000000054> | Study Design |
| Drummond, 2018 | Physicians' real-world experience with IDegLira: results of a European survey  <https://dx.doi.org/10.1136/bmjdrc-2018-000531> | Study Design |
| Ezendu, 2021 | Benefit-Risk Value Assessment of Long-Term Anti-Obesity Drugs | Study Design |
| Gao, 2020 | Barriers to prescribing glucose-lowering therapies with cardiometabolic benefits <https://dx.doi.org/10.1016/j.ahj.2020.03.017> | Study Design |
| Garvey, 2024 | Healthcare professionals' perceptions and management of obesity & knowledge of glucagon, GLP-1, GIP receptor agonists, and dual agonists <https://dx.doi.org/10.1002/osp4.756> | Study Design |
| Gautier, 2015 | Effectiveness and persistence with liraglutide among patients with type 2 diabetes in routine clinical practice—EVIDENCE: a prospective, 2-year follow-up, observational, post-marketing study | Study Design |
| Gourgari, 2021 | Use of glucagon-like peptide-1 receptor agonists for pediatric patients with obesity and diabetes: The providers' perspectives  <https://dx.doi.org/10.1111/pedi.13234> | Study Design |
| Harkin, 2019 | Adjunct liraglutide use in people with type 1 diabetes in the St Vincent's Healthcare Group, Dublin: An Irish perspective | Study Design |
| Hendershot, 2024 | Clinical reports suggest diabetes and obesity drugs like Ozempic and Wegovy may reduce addictive behaviors | Study Design |
| Jain, 2021 | Switching between GLP-1 receptor agonists in clinical practice: Expert consensus and practical guidance  <https://dx.doi.org/10.1111/ijcp.13731> | Study Design |
| Matza, 2016 | Physician perceptions of GLP-1 receptor agonists in the UK  <https://dx.doi.org/10.1185/03007995.2016.1147025> | Study Design |
| Nicolau, 2022 | Short term effects of semaglutide on emotional eating and other abnormal eating patterns among subjects living with obesity  <https://dx.doi.org/10.1016/j.physbeh.2022.113967> | Study Design |
| Qin, 2017 | Glucagon-Like Peptide-1 Receptor Agonist Treatment Attributes Important to Injection-Experienced Patients with Type 2 Diabetes Mellitus: A Preference Study in Germany and the United Kingdom <https://dx.doi.org/10.1007/s13300-017-0237-8> | Study Design |
| Qureshy, 2022 | A survey of physician experience and treatment satisfaction prescribing once-weekly semaglutide injections for patients with type 2 diabetes in Canada <https://dx.doi.org/10.1097/XCE.0000000000000260> | Study Design |
| Sauer, 2015 | Off-label antiobesity treatment in patients without diabetes with GLP-1 agonists in clinical practice <https://dx.doi.org/10.1055/s-0034-1387793> | Study Design |
| Sikirica, 2017 | Reasons for discontinuation of GLP1 receptor agonists: data from a real-world cross-sectional survey of physicians and their patients with type 2 diabetes <https://dx.doi.org/10.2147/DMSO.S141235> | Study Design |
| Spain, 2016 | Self-reported Barriers to Adherence and Persistence to Treatment With Injectable Medications for Type 2 Diabetes <https://dx.doi.org/10.1016/j.clinthera.2016.05.009> | Study Design |
| Sterzi, 2017 | Perceptions of general practitioners on initiation and intensification of type 2 diabetes injectable therapies. A quantitative study in the United Kingdom  <https://dx.doi.org/10.1016/j.pcd.2017.03.003> | Study Design |
| Sterzi, 2017 | Diabetes injectable therapy: type 2 diabetes management in Danish practice  <https://dx.doi.org/10.1080/03007995.2017.1377165> | Study Design |
| van Gorp, 2020 | Insight in the safety profile of antidiabetic agents glucagon-like peptide-1 agonists and dipeptidyl peptidase-4 inhibitors in daily practice from the patient perspective  <https://dx.doi.org/10.1002/pds.5077> | Study Design |
| Yaseen, 2023 | Health Care Provider Prescribing Habits and Barriers to Use of New Type 2 Diabetes Medications: A Single-System Survey Study  <https://dx.doi.org/10.2337/cd22-0100> | Study Design |
